# *Onchocerca lupi* Nematodes in Dogs Exported from the United States into Canada

**DOI:** 10.3201/eid2208.151918

**Published:** 2016-08

**Authors:** Guilherme G. Verocai, Gary Conboy, Manigandan Lejeune, Fany Marron, Paul Hanna, Erin MacDonald, Brian Skorobohach, Brian Wilcock, Susan J. Kutz, John S. Gilleard

**Affiliations:** University of South Florida, Tampa, Florida, USA (G.G. Verocai);; University of Calgary, Calgary, Alberta, Canada (G.G. Verocai, B. Skoroboach, S.J. Kutz, J.S. Gilleard);; Atlantic Veterinary College, Charlottetown, Prince Edward Island, Canada (G. Conboy, F. Marron, P. Hanna);; Canadian Wildlife Health Cooperative, Calgary (M. Lejeune, S.J. Kutz);; Summerside Animal Hospital, Summerside, Prince Edward Island, Canada (E. MacDonald);; Calgary Animal Referral and Emergency Centre, Calgary (B. Skoroboach);; Histovet, Guelph, Ontario, Canada (B. Wilcock)

**Keywords:** Onchocerca lupi, nematode, dogs, canine ocular onchocerciasis, parasites, zoonoses, importation, vector-borne infections, United States, Canada

## Abstract

The *Onchocerca lupi* nematode is an emerging helminth capable of infecting pets and humans. We detected this parasite in 2 dogs that were imported into Canada from the southwestern United States, a region to which this nematode is endemic. We discuss risk for establishment of *O. lupi* in Canada.

*Onchocerca lupi* is species of vectorborne nematode found in dogs, and rarely cats, which was recently recognized as an emerging zoonotic parasite in the United States and Old World countries in Europe and the Middle East (*1**–**3*). Infection in most cases in dogs and cats involves the eyes (*4**–**6*). Incidence of canine cases appears to be increasing; cases have been reported in Germany, Greece, Hungary, Portugal, Romania, Switzerland, and the United States (*2**,**5**,**6*). Only 3 cases of ocular onchocerciasis have been reported in cats: 2 in the United States and 1 in Portugal (*4**,**7*).

Human cases of infection with *O. lupi* nematodes have been reported Old World countries, including Albania, Crimea, Iran, Tunisia, and Turkey (*3**,**8**–**11*). All case-patients had ocular disease caused by subconjunctival nodules containing nematodes. In the United States, these nematodes are emerging zoonotic parasites, and cases are clustered in the southwest, a region to which canine onchocerciasis is endemic. In contrast with Old World zoonotic infections, all human cases in the United States are non-ocular. *O. lupi* nematodes have been found in masses compressing the cervical spinal canal of young children in Arizona and New Mexico (*1**,**11**,**12*). Additional human cases have been found in the same region (*13*).

Little is known about the biology or epidemiology of this emerging zoonotic parasite. As with most *Onchocerca* species, black flies (Simuliidae) serve as biological vectors for *O. lupi*. To date, the only black fly species from which nematode DNA has been isolated is *Simulium tribulatum*, which is endemic to southern California (*5*). We report cases of canine ocular onchocerciasis in Canada.

## The Study

In 2012 and 2014, respectively, 2 privately owned dogs with ocular disease were referred to veterinary practices in Canada. Both animals originated from the southwestern United States.

The first dog (dog A) was from Summerside, Prince Edward Island, in eastern Canada. This dog was a 3-year-old toy fox terrier purchased from a breeder in New Mexico and was taken to Canada months before its death from an unrelated cause (inflammatory bowel disease).

The second dog (dog B) was from Calgary, Alberta in western Canada. This dog was a 7-year-old female pit bull mixed breed that was obtained in Utah 2 years before it developed periodic swelling in the right eye that occurred over ≈1.5 years. This dog underwent surgery for removal of subconjunctival nodules. Two months after surgery, the dog had a friable nodule on the previously healthy left eye. This nodule was also surgically removed.

**Figure 1:**
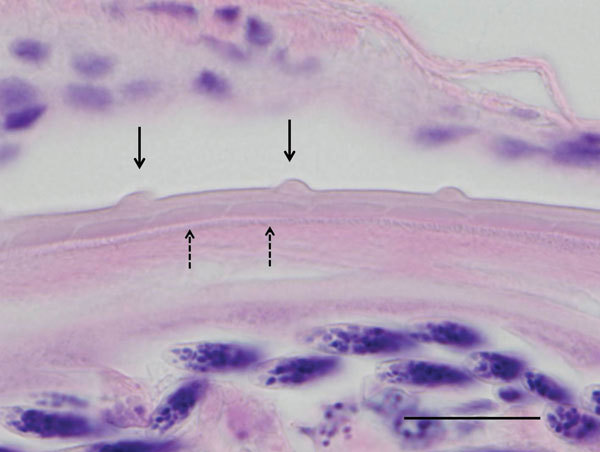
Histologic section of the eye of a dog infected with *Onchocerca lupi* nematodes, Summerside, Prince Edward Island, Canada. The typical *O. lupi* nematode cuticular pattern is shown, with 2 inner transverse striae (dashed arrows) within the interval between 2 outer cuticular ridges (solid arrows). Hematoxylin and eosin stain, original magnification ×100. Scale bar indicates 20 μm.

Adult nematodes were detected in histologic sections of both eyes and microfilaria were detected in skin from an ear of dog A. Nematodes were recovered from surgically removed nodules of dog B. Morphologic examination of nematodes from both dogs showed a cuticular pattern consistent with that of *O. lupi* nematodes, with 2 inner transverse striae per each interval between outer cuticular ridges (Figure 1). Histopathologic analysis of specimens from dog A showed nodular, lymphocytic, granulomatous, eosinophilic conjunctivitis, which is consistent with *O. lupi* nematode infection.

**Figure 2:**
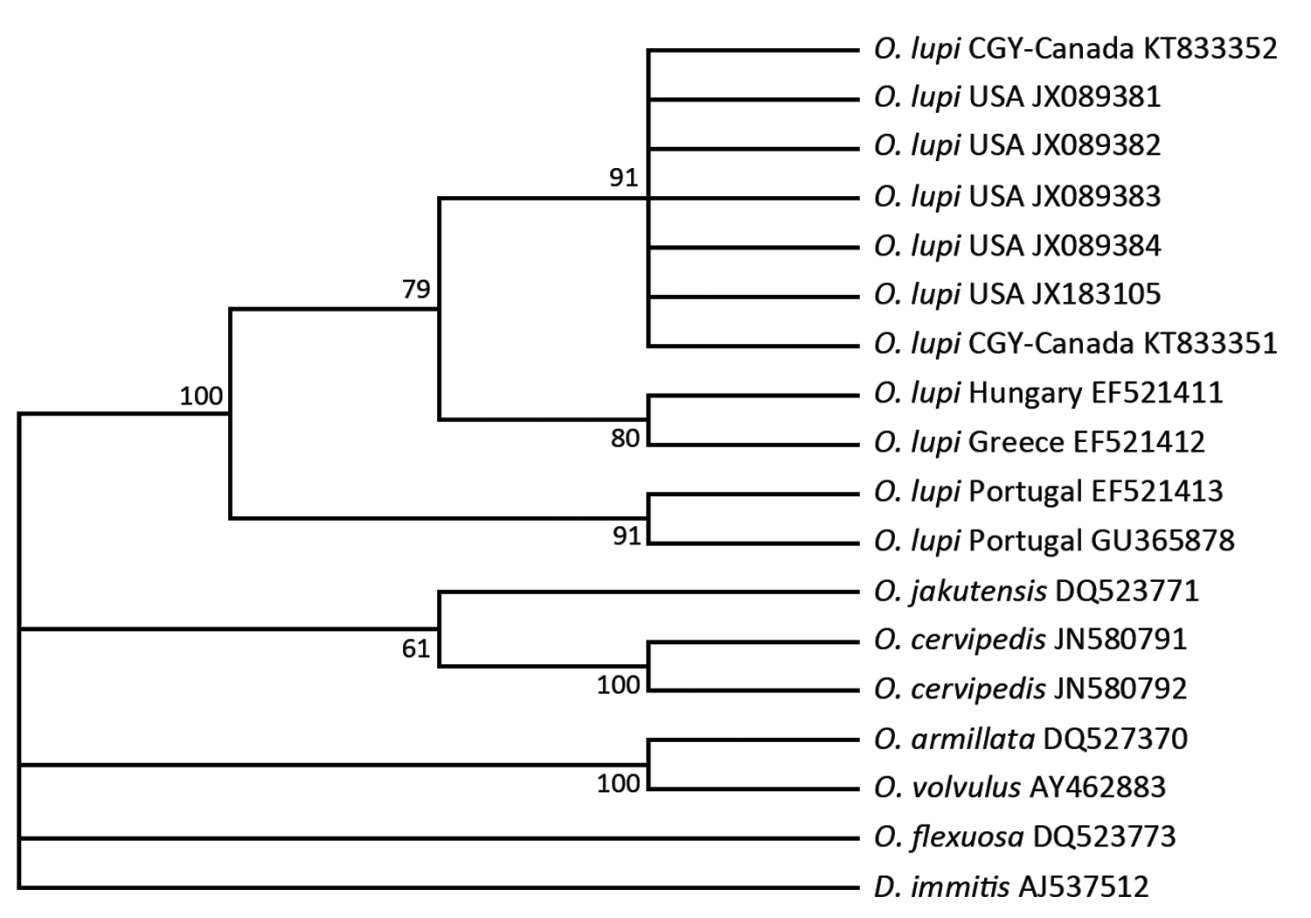
Phylogenetic relationship among the *Onchocerca lupi* nematode isolates from a dog in Calgary, Alberta, Canada (GeneBank accession nos. KT833351 and KT833352), and other filarial nematodes in the family *Onchocercidae* on the basis of the mitochondrial NADH dehydrogenase subunit 5 gene. The parsimonious tree depicts reciprocal monophyly of gene sequence derived from *O. lupi* nematodes from North America and Europe. Bootstrap consensus was inferred from 1,000 replicates. Values along branches are bootstrap values. Branches corresponding to <50% bootstrap replicates are collapsed. GenBank accession numbers are shown for all isolates. Analysis was performed by using MEGA 6 (http://www.megasoftware.net/). Canine heartworm (*Dirofilaria immitis*) was used as an outgroup.

Genomic DNA was extracted from nematode fragments from dog B, and a 420-bp fragment of the mitochondrial NADH dehydrogenase subunit 5 gene was amplified by using PCR and sequenced by using described methods (*14*). Resulting sequences were phylogenetically compared with those of other *O. lupi* nematode isolates from the United States and Europe and other *Onchocerca* species by using MEGA6 (http://www.megasoftware.net/) (Figure 2). Phylogenetic comparison confirmed species identity. All isolates from North America showed 100% similarity and differed by 1–6 bp from isolates from Portugal, Greece, and Hungary.

## Conclusions

We confirmed through molecular and morphological approaches that *O. lupi* nematodes were the causative agent of canine ocular onchocerciasis in 2 dogs in Canada. Both dogs were imported from southwestern United States, which indicates the potential for international dog transportation in contributing to introduction and establishment of zoonotic parasites in nonendemic areas.

The black fly *S. tribulatum*, a putative nematode vector in California (*5*), is widely distributed across the United States and Canada, including areas of southern Alberta, where Calgary is located (*15*). This fly is one of many species in the *S. vittatum* species complex and is also found in Prince Edward Island, Canada (*15*). The wide occurrence of the confirmed vector and related potential vectors in these areas of Canada and many areas of the United States reinforce the need for diagnosis and treatment of onchocerciasis before importation of dogs from disease-endemic areas.

The biology of *O. lupi* nematodes is poorly understood. However, theses nematodes appear to have a long life span. Consequently, microfilariae might be available for long periods (a few years) in skin of infected dogs. Available microfilariae might be ingested by competent black fly vectors, which will feed on the ubiquitous vertebrate hosts for *O. lupi* nematodes (e.g., dogs, cats, and humans) and thus pose a risk for establishment of the parasite in areas where it has been introduced. Clinical ocular cases are believed to occur in only a small portion of overall canine infections (*6*). Nonclinical cases, together with rare nonocular clinical cases, in dogs and potential wildlife reservoirs (e.g., wolves, coyotes) might make a larger contribution to the epidemiology of canine onchocerciasis.

Reappearance of an *O. lupi* nodule in the previously healthy eye of dog B ≈3 months after surgical removal of the first nodules remains enigmatic. Presumably, the second nodule was also caused by exposure in the United States. It seems unlikely that during the 2 years spent in Canada, dog B served as a source of infection for the local black flies, which would have led to a second exposure of the same dog in Canada. We are not aware of any reports of local cases. Both dogs probably had long-term patent infections while in Canada. As adult dogs of active breeds, with a history of outdoor activities and travel, these 2 dogs might have been exposed to black flies during months of optimal environmental conditions for these vectors. Dog A traveled frequently to other parts of Canada and the United States as a show dog, including multiple trips in the spring/summer months.

A recent study hypothesized that *O. lupi* nematodes were recently introduced to the United States, possibly by dogs from Europe (*6*). Similarly, moving infected animals to areas of the United States and Canada to which *O. lupi* nematodes are not endemic might facilitate range expansion. Because purchase or adoption of pets from the United States is a common practice in Canada, additional clinical and nonclinical cases of canine onchocerciasis might be present in Canada, which would increase the risk for establishment of this zoonotic parasite.

Currently, diagnosis in dogs before onset of ocular disease would require recovery of microfilariae from a skin biopsy specimen. The unfamiliarity of diagnostic laboratories in North America with such testing and its unknown sensitivity make it unlikely that current infrastructure could effectively screen the number of dogs crossing the border to prevent introduction. The only current requirement for dog and cat importation into Canada by the Canadian Food Inspection Agency is having an up-to-date rabies immunization (http://www.inspection.gc.ca/animals/terrestrial-animals/imports/policies/live-animals/pets/eng/1326600389775/1326600500578). Because suitable intermediate and definitive hosts of *O. lupi* nematodes are already present in Canada, there is an ongoing risk for the nematode to become established, which might be dependent on climatic and ecologic factors.

## References

[1] Eberhard ML, Ostovar GA, Chundu K, Hobohm D, Feiz-Erfan I, Mathison BA, Zoonotic *Onchocerca lupi* infection in a 22-month-old child in Arizona: first report in the United States and a review of the literature. Am J Trop Med Hyg. 2013;88:601–5. 10.4269/ajtmh.12-0733 23382171 PMC3592550

[2] Grácio AJ, Richter J, Komnenou AT, Grácio MA. Onchocerciasis caused by *Onchocerca lupi*: an emerging zoonotic infection. Systematic review. Parasitol Res. 2015;114:2401–13. 10.1007/s00436-015-4535-7 25990062

[3] Otranto D, Dantas-Torres F, Cebeci Z, Yeniad B, Buyukbabani N, Boral OB, Human ocular filariasis: further evidence on the zoonotic role of *Onchocerca lupi.* Parasit Vectors. 2012;5:84. 10.1186/1756-3305-5-84 22541132 PMC3407723

[4] Labelle AL, Daniels JB, Dix M, Labelle P. *Onchocerca lupi* causing ocular disease in two cats. Vet Ophthalmol. 2011;14:105–10. 10.1111/j.1463-5224.2011.00911.x 21923832

[5] Hassan HK, Bolcen S, Kubofcik J, Nutman TB, Eberhard ML, Middleton K, Isolation of *Onchocerca lupi* in dogs and black flies, California, United States. Emerg Infect Dis. 2015;21:789–96. 10.3201/eid2105.142011 25897954 PMC4412245

[6] Otranto D, Giannelli A, Latrofa MS, Dantas-Torres F, Trumble NS, Chavkin M, Canine infections with *Onchocerca lupi* nematodes, United States, 2011–2014. Emerg Infect Dis. 2015;21:868–71. 10.3201/eid2105.141812 25897859 PMC4412234

[7] Maia C, Annoscia G, Latrofa MS, Pereira A, Giannelli A, Pedroso L, *Onchocerca lupi* nematode in cat, Portugal. Emerg Infect Dis. 2015;21:2252–4. 10.3201/eid2112.150061 26584050 PMC4672443

[8] Ilhan HD, Yaman A, Morishima Y, Sugiyama H, Muto M, Yamasaki H, *Onchocerca lupi* infection in Turkey: a unique case of a rare human parasite. Acta Parasitol. 2013;58:384–8. 10.2478/s11686-013-0152-8 23990437

[9] Otranto D, Sakru N, Testini G, Gurlu VP, Yakar K, Lia RP, Case report: first evidence of human zoonotic infection by *Onchocerca lupi* (Spirurida, Onchocercidae). Am J Trop Med Hyg. 2011;84:55–8 . 10.4269/ajtmh.2011.10-0465 21212202 PMC3005520

[10] Mowlavi G, Farzbod F, Kheirkhah A, Mobedi I, Bowman DD, Naddaf SR. Human ocular onchocerciasis caused by *Onchocerca lupi* (Spirurida, Onchocercidae) in Iran. J Helminthol. 2014;88:250–5. 10.1017/S0022149X13000060 23388686

[11] Chen T, Moon K, deMello DE, Feiz-Erfan I, Theodore N, Bhardwaj RD. Case report of an epidural cervical *Onchocerca lupi* infection in a 13-year-old boy. J Neurosurg Pediatr. 2015;16:217–21. 10.3171/2014.12.PEDS14462 25932778

[12] Dudley RW, Smith C, Dishop M, Mirsky D, Handler MH, Rao S. A cervical spine mass caused by *Onchocerca lupi.* Lancet. 2015;386:1372. 10.1016/S0140-6736(14)62255-8 25843892

[13] Cantey PT, Weeks J, Edwards M, Rao S, Ostovar GA, Dehority W, The emergence of zoonotic *Onchocerca lupi* infection in the United States: a case-series. Clin Infect Dis. 2016;62:778–83. 10.1093/cid/civ983 26611778 PMC4809994

[14] McFrederick QS, Haselkorn TS, Verocai GG, Jaenike J. Cryptic *Onchocerca* species infecting North American cervids, with implications for the evolutionary history of host association in *Onchocerca.* Parasitology. 2013;140:1201–10. 10.1017/S0031182012001758 23131549

[15] Adler PH, Currie DC, Wood M. The black flies (Simuliidae) of North America. New York: Comstock Books; 2004.

